# miR-143 Interferes with ERK5 Signaling, and Abrogates Prostate Cancer Progression in Mice

**DOI:** 10.1371/journal.pone.0007542

**Published:** 2009-10-26

**Authors:** Cyrielle Clapé, Vanessa Fritz, Corinne Henriquet, Florence Apparailly, Pedro Luis Fernandez, François Iborra, Christophe Avancès, Martin Villalba, Stéphane Culine, Lluis Fajas

**Affiliations:** 1 IRCM, Institut de Recherche en Cancérologie de Montpellier, Montpellier, France; 2 INSERM, U896, Montpellier, France; 3 Université de Montpellier1, Montpellier, France; 4 Centre Regional de Lutte contre le Cancer Val d'Aurelle Paul Lamarque, Montpellier, France; 5 INSERM U844, Montpellier, France; 6 Institut d'Investigacions Biomèdiques August Pi i Sunyer, Barcelona, Spain; 7 Department of Pathology, Hospital Clínic de Barcelona, Barcelona, Spain; 8 Service d'urologie, Centre Hospitalier Universitaire Lapeyronie, Montpellier, France; 9 Service d'Urologie, CHU Groupe Hospitalisation Carémeau, Nîmes, France; 10 Service d'Urologie, Polyclinique Kennedy, Nîmes, France; 11 Institut de Génétique Moléculaire, Montpellier, France; 12 CNRS, UMR5535, Montpellier, France; 13 Université Montpellier2, Montpellier, France; 14 Department of Medical Oncology, CHU Henri Mondor, Creteil, France; Baylor College of Medicine, United States of America

## Abstract

**Background:**

Micro RNAs are small, non-coding, single-stranded RNAs that negatively regulate gene expression at the post-transcriptional level. Since miR-143 was found to be down-regulated in prostate cancer cells, we wanted to analyze its expression in human prostate cancer, and test the ability of miR-43 to arrest prostate cancer cell growth in vitro and in vivo.

**Results:**

Expression of miR-143 was analyzed in human prostate cancers by quantitative PCR, and by in situ hybridization. miR-143 was introduced in cancer cells in vivo by electroporation. Bioinformatics analysis and luciferase-based assays were used to determine miR-143 targets. We show in this study that miR-143 levels are inversely correlated with advanced stages of prostate cancer. Rescue of miR-143 expression in cancer cells results in the arrest of cell proliferation and the abrogation of tumor growth in mice. Furthermore, we show that the effects of miR-143 are mediated, at least in part by the inhibition of extracellular signal-regulated kinase-5 (ERK5) activity. We show here that ERK5 is a miR-143 target in prostate cancer.

**Conclusions:**

miR-143 is as a new target for prostate cancer treatment.

## Introduction

Prostate cancer (CaP) is the most frequent cancer and the second leading cause of cancer death in men in western countries. Androgen receptor (AR) is likely a crucial factor in prostate cancer progression. Prostate cancer is initially dependent on androgens for growth, and androgen ablation therapy causes regression of the tumor [1], likely through inactivation of the transcription of the AR target genes. However, response to this therapy is often transient and many men develop recurrent androgen-independent prostate cancer, which has a very poor prognosis because no effective treatment is currently available (see [2] for review).

Recently, a new class of small RNAs has been described, termed microRNAs, which are found to regulate mRNA function by modulating both mRNA stability and the translation [3], [4]. MiRNAs are small, non-coding, single-stranded RNAs of ∼22 nucleotides that negatively regulate gene expression at the post-transcriptional level, primarily through base pairing to the 3′ untranslated region (UTR) of target mRNAs. Growing evidence indicates that miRNAs control basic cell functions, ranging from proliferation to apoptosis [5], [6], [7]. Approximately 50% of miRNA genes are located in cancer-associated genomic regions or in fragile sites [8] and some of them have been shown to be directly involved in cancer development and progression [9]. MicroRNA expression profiles also classify tumors by developmental lineage and differentiation state [10]. Multiple microRNAs have been shown to have oncogenic properties or act like tumor suppressor genes [9], [11]. This is the case for miR-15A and miR-16-1, which expression is lost in advanced prostate cancer. These two miRNAs show anti-oncogenic activity through targeting of cyclin D1 and Wnt3A, which are mediators of cancer cell proliferation and survival [12]. Decreased expression of other miRNAs, such as miR-23b, -100, -145, -221 and -222 has been also documented. Moreover, ectopic expression of these miRNAs results in prostate cancer cell inhibition [13]. Contrary to these anti-oncogenic miRNA, oncogenic miR-106b, or miR-32 have been identified in prostate cancer. These miRNA support cell proliferation and survival of cancer cells trough targeting of p21/WAF1 and Bim proteins respectively [14]. Most interestingly is the observation that some miRNA, such as miR-141 are secreted by cancer cells, and are found in serum of prostate cancer patients. These results establish the measurement of tumor-derived miRNAs in serum or plasma as a novel diagnostic tool for a non-invasive method of detection of human cancer [15].

We have previously shown that a combination treatment using PPARγ agonists and HDAC inhibitors results in the inhibition of prostate cancer cell growth in mice [16]. Global analysis of micro RNA expression in these treated cells correlated increased miR-143 with growth arrest. In the present study we investigated the expression of miR-143 in prostate cancer and found that the expression level of miR-143 was significantly decreased. Furthermore, the expression level was inversely correlated with histopathological grade in human prostate cancer. Transfection of LNCaP and C4-2 cells in vitro with precursor miR-143, or electroporation of miR-143 in prostate cancer xenografts in mice demonstrated that miR-143 negatively contributes to prostate cancer cell growth. Finally, bioinformatics analyses identified ERK5 as a potential target gene for mir-143. ERK5 is known to promote cell growth and proliferation in response to growth factors and tyrosine kinase activation. Therefore, persistent decreased levels of mir-143 in cancer cells may be directly involved in carcinogenesis through activation of the mitogen-activated protein kinase (MAPK) cascade via ERK5. Taken together these findings suggest that mir-143 could be a tumor suppressor and a potential novel diagnostic or prognostic marker in prostate cancer.

## Results

### miR-143 expression is decreased during prostate cancer progression

**Figure 1:**
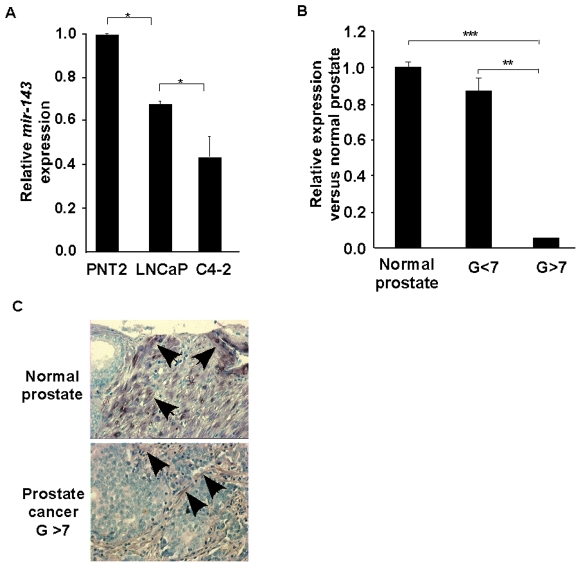
Expression of miR-143 in prostate cancer. *A*, Quantitative real-time PCR (QPCR) of miR-143, normalized to the amount of RNU48 target in prostate cancer cell lines. The relative levels of miRNA expression were measured by determining the ΔCt values of the indicated cell line versus PNT2 cells. Data are means ± SEM for three independent experiments. Statistical significance, here and in subsequent figures, *<0,05; **<0,01; ***<0,001. *B*, QPCR of miR-143, normalized to the amount of RNU48 target in prostate tissue samples. The relative levels of miRNA expression were measured by determining the ΔCt values of the indicated gleason prostate cancer versus normal prostate. Data are means ± SEM of eleven prostate samples for each group. *C*, Representative *in situ* hybridization staining of miR-143 in human non-tumor and tumor prostate tissue. TMA presents 40 prostate cancer tissues vs. 10 normal prostate tissues. (TMA, magnification, 400×).

A first indication of the participation of miR-143 in prostate cancer was the observed decreased expression of this miRNA in LNCaP, and C4-2 prostate cancer, compared to normal epithelial cell lines, as analyzed by quantitative RT-PCR (Fig. 1A). Consistent with this observation, the expression of miR-143 was strongly decreased in human prostate cancer, compared to normal prostate tissues (fig. 1B). Furthermore, levels of transcribed miR-143 were inversely correlated with histopathological grade in human prostate cancer, reaching the limit of detection in high-grade cancers (Gleason >7; Fig. 1B). To more precisely analyze expression of miR-143, *in situ* hybridization was performed in high-density tissue arrays, containing 40 prostate cancer tissues vs. 10 normal prostate tissues. miR-143 expression could not be detected specifically in prostate cancer cells of high Gleason score, whereas miR-143 was expressed in normal prostate epithelium, and prostatic glands (Fig. 1C). These results suggested that downregulation of miR-143 expression could be a required event for cancer development or progression.

### Decreased proliferation and increased apoptosis in miR-143-expressing prostate cancer cells

**Figure 2:**
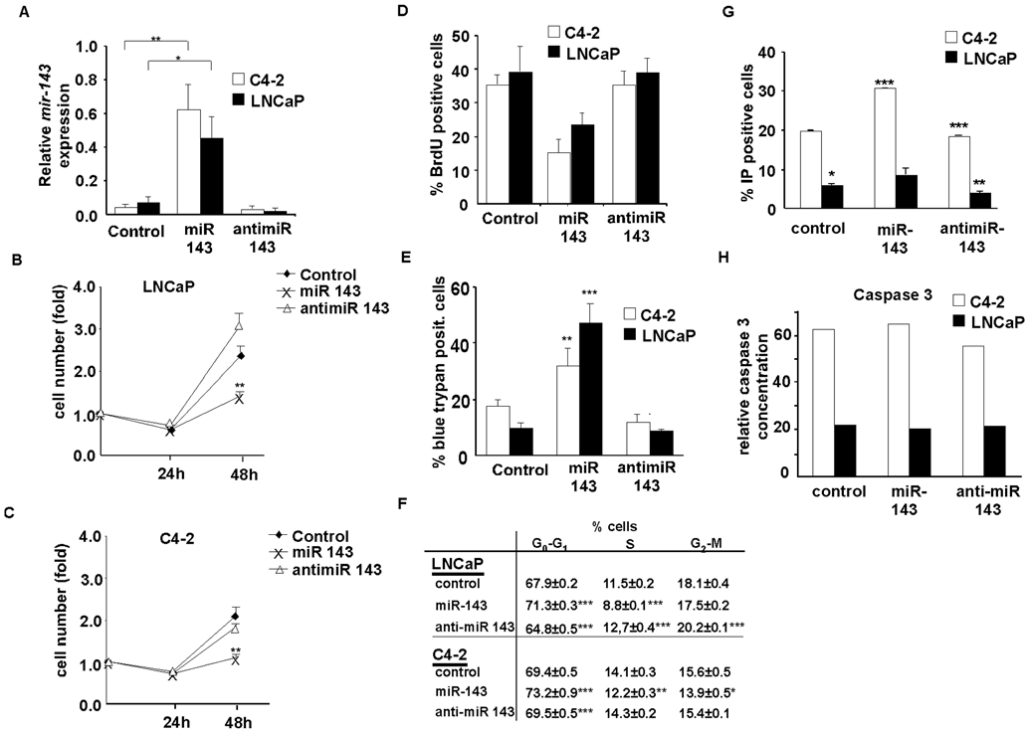
miR-143 overexpression in C4-2 cells and LNCaP cells. *A*, QPCR of miR-143 in C4-2 cells (white bars) and LNCaP cells (black bars), normalized to RNU48 48 h after transfection with scrambled miR (control), miR-143 precursor or antimiR-143. Data are means ± SEM for five independent experiments. *B–C*, Cell growth in LNCaP (B) or C4-2 (C) cells during 48 h after transfection with scrambled miR (black rhombus), miR-143 precursor (black cross) or antimiR-143 (white triangle). Data are means ± SEM for three independent experiments. *D*, Quantification of BrdU incorporation 48 h after transfection in C4-2 and LNCaP cells in absence of miR-143 (top), in presence of miR-143 (middle) or antimiR-143 (bottom). Data are representative for three independent experiments. *E*, Blue trypan incorporation in C4-2 (white bars) and LNCaP (black bars) cells 48 h after transfection in absence or presence of miR-143 or in presence of antimiR-143. *F*, Percentage of cells in different phases of cell cycle in LNCaP and C4-2 cell lines 36 hours following transfection with control 5 (non relevant), miR-143 or antimiR-143. Similar results were obtained in two independent experiments. Results are expressed as mean ± sem (n = 2–4). *G*, FACS analysis of apoptosis in C4-2 (white bars) and LNCaP (black bars) cells 48 h after transfection in absence or presence of miR-143 or in presence of antimiR-143. *H*, Relative active Caspase 3 concentration in C4-2 (white bars) and LNCaP (black bars) cells 48 h after transfection in absence or presence of miR-143 or in presence of antimiR-143. The concentration of active caspases 3 is normalized with the global protein concentration.

Decreased expression in prostate cancer suggested that miR-143 could have anti-proliferative effects. To test this hypothesis miR-143 was ectopically expressed in C4-2 and LNCaP prostate cancer cell lines. miR-143 was expressed 5- and 12-fold higher in transfected LNCaP, and C4-2 cells respectively, compared to control cells transfected with either non-relevant miRNA, or with anti-miR-143, which is a miR-143 inhibitor (Fig. 2A). No differences in cell numbers were observed when non-relevant miRNA or anti-miR-143 were used in these cells. In sharp contrast, cell number was inversely correlated to the expression levels of miR-143 in both LNCaP, and C4-2 cell lines (Fig. 2B–C). This suggested that miR-143 negatively regulates cell proliferation. Consistent with this, BrdU incorporation experiments proved that miRNA expression results in the inhibition of DNA synthesis, and therefore abrogation of cell proliferation in these cancer cell lines (Fig 2D). Interestingly, these cytostatic effects were also correlated with increased cell death in miRNA expressing prostate cancer cells, as evaluated by blue trypan experiments (Fig. 2E). Furthermore, cell cycle analysis showed an increase in the G_0_-G_1_ population, concomitant to a decrease in S-phase in miR-143 expressing cells (Fig. 2F). Interestingly, FACS analysis indicated an increase in cell death (Fig. 2G). Apoptosis was, however, not changed in miR-143, compared to control cells as assessed by annexine incorporation experiments (data not shown), or by caspase 3 level 48 h after transfection (Fig. 2H). This suggested that the observed cell death is not dependent on apoptosis, but rather necrosis events. Altogether, these data suggested that miR-143 negatively controls cell proliferation and positively controls cell death of prostate cancer cells.

### ERK5 is a miR-143 target gene

**Figure 3:**
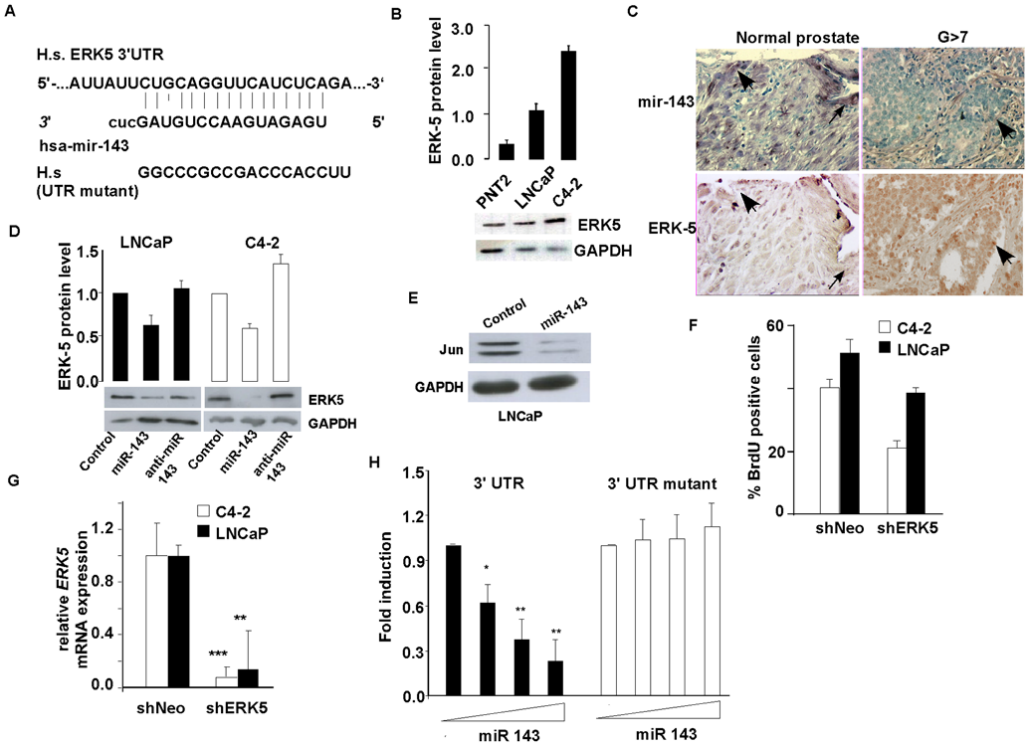
Identification of ERK5 as a miR-143 target. *A*, Schematic representation of the predicted target site of miR-143 in the 3′ UTR of ERK5 mRNA. Seed-matching sequence is indicated. H.s ERK5 3′UTR is the Homo Sapiens 3′UTRsequence. *B*, Western blot analysis of endogenous ERK5 expression in prostate normal and cancer cell lines. The relative expressions, normalized to GAPDH, were measured by Image J software as a fold of the indicated situation versus scrambled miR. Data are means ± SEM for three independent experiments. C, Representative immunohistochemical staining of ERK5, and in situ hybridization of miR-143 in consecutive sections of high-density tissue array. *D*, Western blot analysis of endogenous ERK5 expression in C4-2 and LNCaP cells 48 h after transient transfection of the indicated miR-143. Relative expression, quantified by Image J software is normalized to GAPDH, and measured by fold of the indicated situation versus scrambled miR. Data are means ± SEM for three independent experiments. *E*, Measure of the luciferase activity in COS cells transfected with a reporter luciferase gene pGL3 fused to the ERK-5 3′ UTR mutated or not with increasing concentrations or miR-143 (0; 25; 50; 100 nM). Values are normalized to beta-galactosidase activity and expressed in fold versus absence of miR-143. Black bars, ERK5 3′UTR, white bars, mutant 3′ UTR; data are means ±SEM for four experiments conducted in triplicate. *F*, Western blot analysis of endogenous c-Jun expression in LNCaP cells 48 h after transient transfection of the indicated miR-143. Data are representative for three independent experiments. *G*, QPCR of ERK5 in C4-2 cells (white bars) and LNCaP cells (black bars), normalized to 18 s gene 48 h after transfection with pSuper-shNeo (control) or pSuper-shERK5. Data are means ± SEM for three independent experiments. *H*, Quantification of BrdU incorporation in C4-2 cells (white bars) and LNCaP cells (black bars) 48 after transfection with pSuper-shNeo (control) or pSuper-shERK5. Data are representative for three independent experiments.

We wanted to identify miR-143 targets that could be implicated in prostate cancer progression. Bioinformatics analysis indicated that the 3′ UTR of the human ERK-5 gene harbors a putative consensus site for miR-143 binding (nucleotides 2917-2932) (fig 3A). To experimentally validate this target, we first analyzed ERK5 expression in prostate cancer cell lines. ERK5 protein was highly increased in LNCaP, and C4-2 prostate cancer cells, compared to non-cancerous PNT2 prostatic epithelial cells (Fig. 3B). Interestingly, ERK5 expression was inversely correlated with miR-143 expression in these cell lines (Fig. 1A). Further analysis in human prostate cancer in high-density tissue arrays showed that ERK5 expression, as analyzed by IHC was highly increased in cancer, compared to normal prostate tissue (Fig 3C, lower panels). Strikingly, ERK5 expression was inversely correlated with miR-143 expression, as analyzed by in situ hybridization, suggesting that ERK5 could be a *bona fide* miR-143 target (Fig. 3C, compare upper to lower panels). To prove this hypothesis overexpression experiments were performed. Ectopic expression of miR-143 in LNCaP, and C4-2 prostate cancer cell lines resulted in a significant decrease in ERK5 protein expression, compared to cells transfected with non-relevant miRNA, or with the antisense miR-143. (Fig. 3D). Decreased ERK5 expression correlated with decreased proliferation in these cell lines upon expression of miR-143 (Fig. 2). Moreover, decreased ERK 5 expression in mir-143 treated cells also correlated with decreased expression of Jun, which is a known ERK5 target (Fig. 3E).

Next, to further demonstrate the hypothesis that the observed effects on proliferation are the result of ERK5 inhibition, silencing experiments were performed. shRNA expression directed to ERK5 resulted in a significant decrease in cell proliferation in LNCaP and C4-2, compared to cells transfected with non-relevant shRNA (Fig. 3F). ERK5 expression level was decreased about 90% in both cell lines transfected with the specific shERK5 (Fig. 3G).

Finally, to further prove that ERK5 is a miR-143 target gene transient transfection experiments were performed using the 3′UTR region of ERK5 containing the putative miR-143 matching site, mutated or not, downstream of the luciferase open reading frame. Luciferase activity of the 3′ UTR ERK-Luc construct was decreased in the presence of miR-143, whereas luciferase activity of mutated -Luc construct was not affected, suggesting that miR-143 modulate ERK5 expression through binding to ERK5-3′UTR (fig 3F).

### Rescue of miR-143 expression decreases tumor progression in mice

**Figure 4:**
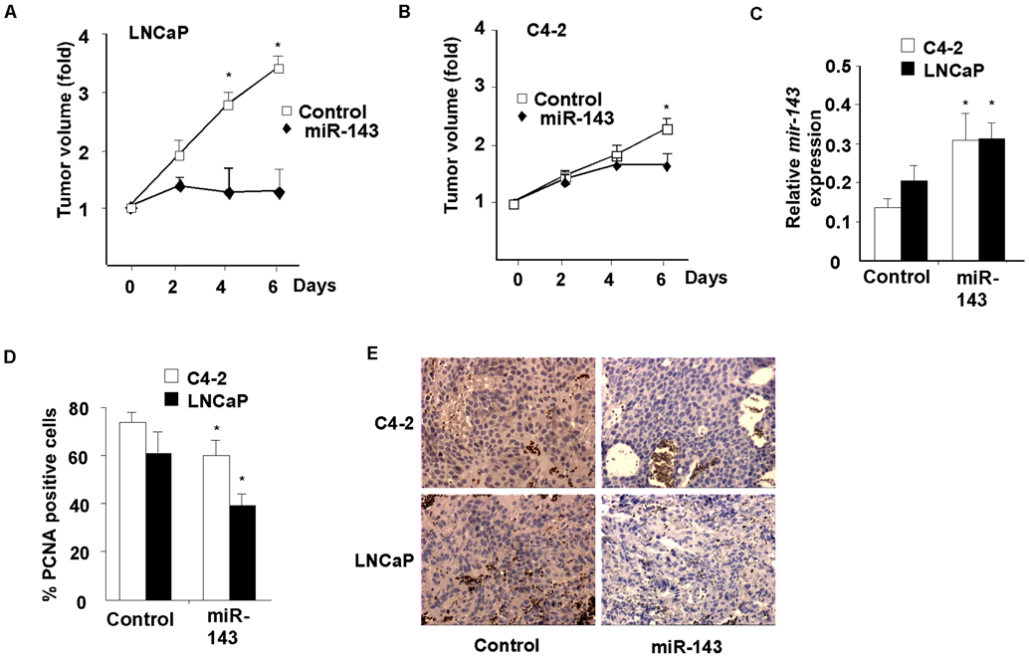
*E*ffects of miR-143 rescue in mice model of prostate tumors. *A–B*, Tumor growth of LNCaP (A) or C4-2 (B) cells injected subcutaneously and electroporated three times with non-relevant miR (control, white squared) or miR-143 precursor (black rhombus). Data are means ± SEM for seven mice analyzed per each group. *C*, QPCR analysis of miR-143 expression in C4-2 (white bars) and LNCaP xenografts (black bars), normalized to RNU48. Data are means ± SEM for seven mice analyzed per each group. *D*, Analysis of cell proliferation by PCNA staining on xenograft sections of athymic Nude mice injected subcutaneously with C4-2 (white bars) or LNCaP (black bars) cells after three electroporations with scrambled miR or miR-143 precursor. Data are means ± SEM for seven mice analyzed per each group. *E*, Representative immunohistological staining of ERK5 in C4-2 and LNCaP tumors. Nude mice injected subcutaneously with C4-2 (white bars) or LNCaP (black bars) cells after three electroporations with scrambled miR or miR-143 precursor. Data are representative for seven mice analyzed per each group. (magnification, 400×).

To investigate the effect of miR-143 on tumor growth, athymic nude mice were subcutaneously grafted with prostate cancer LNCaP and C4-2 cells. Two weeks after inoculation of the cells, when tumors reached a mean volume of 392 mm^3^, miR-143 rescue experiments were performed. Intratumoral injection of miR-143, followed by electroporation as described in material and methods section resulted in the abrogation or decrease in tumor growth in mice grafted with LNCaP and C4-2 cells respectively (Fig. 4A and 4B), whereas electroporation of control non-relevant miR had no effect on tumor growth (Fig. 4A and 4B). In spite of the expected miR-143 rapid degradation in vivo, expression of miR-143 remained significantly increased in miR-143 electroporated tumors (Fig. 4C). Consistent with the observed decrease in tumor growth, the proliferation ratio of LNCaP and C4-2 tumors was also decreased, as quantified by PCNA staining in tumors electroporated with miR-143 (Fig. 4D). Finally, analysis of ERK5 protein level by immunohistochemistry indicated that ERK5 expression was decreased in presence of miR-143 in LNCaP and C4-2 tumors (Fig. 4E). Taken together these results proved that miR-143 functions as a tumor suppressor in prostate cancer cells.

## Discussion

We have analyzed the expression and the effects of miR-143 on prostate cancer development and progression. Some studies have previously shown that miR-143 could play a role in tumorigenesis since its expression is decreased in several cancers [19]. We report two major findings in this study. First, we show that miR-143 expression is clearly downregulated during prostate cancer progression. Screening strategies have resulted in the detection of prostate cancer at progressively earlier stages and lower levels of prognostic risk [20]. In many men, prostate cancer have a long natural history, while others will progress to metastatic stage and die from their disease (reviewed in [21]). Despite the accepted value of measuring prostate-specifc antigen (PSA) levels to screen for prostate cancer diagnosis, its value as a prognostic marker is still a matter of debate. Other tumour-related parameters including clinical staging using the TNM system, Gleason biopsy score and pretreatment serum prostate-specific antigen (PSA) level are currently used to classify patients as being at low-, intermediate- or high-risk for development of aggressive cancer [22]–[24]. No single analysis is able, however to adequately identify all patients with localized prostate cancer that have a high likelihood of progression after therapy. Our finding that miR-143 is at the limit of detection in aggressive prostate cancer could help to identify such patients. Consistent with our findings expression of miR-143 has been found to be decreased also in other cancers, such as colon cancer [25], B-cell malignancies [26], prostate cancer [27], pituitary tumors [28], or cervical cancer [29]. This down-regulation of miR-143 in prostate cancer samples has been suggested to reflect the lower differentiation stage of the tumor tissue compared the normal tissue [30]. Further prospective studies to validate miR-143 as a predictive target of prostate cancer progression are warranted.

The second major finding of our study is the observation that rescue of miR-143 expression in prostate cancer cells results in the abrogation of cancer cell growth, both in vitro and in mice. We proved, for the first time that miR-143 is a tumor suppressor in mice. Other studies have pointed to a tumor suppressor role of miR-143 in colon, and other cancer cells [26], particularly through inhibition of KRAS, and ERK5 proteins [26], [31]. It has been shown that ERK5 might be a direct or indirect target of miR-143 [32]. We show now and we provide enough evidence to demonstrate that ERK5 is a direct target of miR-143 in prostate cancer. This is supported by the presence of miR-143 binding site in the 3′UTR of ERK5 mRNA. Furthermore, miR-143 inhibits expression of a reporter protein when this binding site replaces the 3′UTR of the luciferase mRNA. Finally, expression of ERK5 protein is inversely correlated to miR-143 expression in human prostate cancers. ERK5 has been implicated in the regulation of cell proliferation, and is the most recently identified MAPKK [33]. The activities of several transcription factors, mostly implicated in cancer have been shown to be regulated by ERK5, including MEF2, c-Fos and Fra1, Sap-1, c-Myc and NF-kappaB [34]–[37]. Consistent with our results ERK5 overexpression is associated with metastatic prostate cancer and induces proliferation, motility and invasion in prostate cancer cells [38].

In summary we have shown that miR-143 is a tumor suppressor miRNA in prostate cancer, that controls cell proliferation and survival through modulation of, at least in part ERK5 expression. Rescue of miRNA expression in prostate tumors should be therefore considered as new target for prostate cancer treatment.

## Materials and Methods

### Ethics statement

All human samples were purchased. Our providers Biochain (Hayward, CA), Cytomix (Lexington, MA) have the required permissions.

### Clinical samples

Thirty-seven non-paired (13 normal and 24 tumor RNAs from different patients) and one paired prostate tumor and its surrounding unaffected tissue RNAs were obtained from Biochain (Hayward, CA), Cytomix (Lexington, MA).

### Animals., and in vivo electroporation

Male athymic nude Mice (Foxn1 nu/nu) (Harlan, Grannat, France) were used at the age of 7 weeks. All procedures were performed in compliance with the European Convention for the Protection of Vertebrate Animals Used for Experimentation and approved by the Comite d'Ethique du IRCM de Montpellier, which is the local ethics committee. Xenografts were established by subcutaneously injecting 2×10^6^ LNCaP or C4-2 cells in 100 ul of a Matrigel solution. Tumor volume measurements were taken every two days just before electroporation. At euthanasia, tumors were excised and either frozen for RNA extraction or fixed in 4% formalin for immunohistological analysis. For intra-xenograft electrotransfer, mice were anaesthetized by intra-peritoneal injection of a ketamine (30 mg/Kg) and xylazine (10 mg/Kg) solution. 500pmole of miR-143 precursor or scrambled miR were injected in 100 µL PBS into the xenograft. Before injections, echographic gel was applied, transcutaneous electric pulses were applied using two custom-made stainless steel plate electrodes placed on either side of the xenograft as previously described (Takei et al., 2008). Briefly, 8 square wave electric pulses of 50 msec length, and an output voltage of 100 V/cm were generated by an electropulsator ECM-830 (BTX, San Diego, CA, USA).

### Cell culture and transfections

PNT2 prostate epithelial cell line was obtained from ECACC (Sigma-Aldrich, Lyon, France). The androgen-sensitive LNCaP and the androgen-independent C4-2 human prostate carcinoma cell lines were purchased from ViroMed Laboratories (Minnetonka, MN). These cell lines were maintained as previously described [16]. Transfections with miR-143 precursor, anti-miR 143 (hsa-mir-143, Ambion) were performed at concentration of 50 nM with Dharmafect 2 (Dharmacon, Lafayette, CO). The control is a non-relevant miR precursor, which will be matured in a stable form by the RNAi machinery. The anti-miR-143 is able to inhibit miR-143 present in cells by hybridization. To knockdown ERK5 protein level transfections were performed with 2 µg plasmid pSuper-shERK5 or with a negative control, pSuper-shNeo with JetPEI transfectant. After 48 h, cells were treated with BrdU and then fixed with 4% paraformaldehyde for further analysis.

### RNA isolation, reverse transcription and quantitative real-time PCR

Total RNA and QPCR experiments were performed as described [17].The oligonucleotide sequences used for various experiments in this manuscript are available upon request.

### Quantitative Real-Time PCR for mature microRNA

cDNA was reverse transcribed from 10 ng of total RNA samples using specific primers from Taq Man MicroRNA Assays and reagents from the Taq Man MicroRNA Reverse Transcription kit (Applied Biosystems). The resulting cDNA was amplified by PCR using Taq Man MicroRNA Assay primers with Taq Man universal PCR Master Mix and analyzed with a 7300 ABI PRISM Sequence Detector System according manufacture's instructions (Applied Biosystems). Using the comparative CT method, we used endogenous control (RNU48) to normalize the expression levels of miRNA. The assay names for miR-143 and U48 were as follows: hsa-mir-143 for miR-143 and RNU48 for U48 RNA.

### MicroRNA target prediction

We used miRBase Targets (http://microrna.sanger.ac.uk/targets/v5/) and TargetScan (http://www.targetscan.org/) for microRNA target prediction.

### miR Reporter Luciferase Assays

The 3′UTR or mutant 3′UTR of ERK5 were cloned in the XbaI site of the pGL3-Control Vector, immediately downstream of the stop codon of luciferase coding sequence. COS cells were co-transfected with 0.5 µg of pGL3-based vectors and different concentrations of miR-143 precursor as indicated, using Lipofectamine 2000. Luciferase activity measurements were normalized for β-galactosidase activity to correct for differences in transfection efficiency.

### Immunoblots

Protein extraction, and western blot analyses were performed as previously described [18]. Filters were incubated overnight at 4°C with rabbit anti-ERK5 antibody (Cell Signalling Technology, Danvers, MA) and then for 1 h at room temperature with an anti-rabbit peroxidase-conjugate secondary antibody. The complex was visualized with enhanced chemiluminescence (Interchim, Montluçon, France).

### Immunohistochemistry of ERK5

Tissue Arrays (TMA) AccuMax Array containing 7 normal and 39 adenocarcinoma spots from different patients were obtained from Alphelys (Plaisir, France). For immunohistochemical staining, TMAs were depleted of paraffin with xylene, rehydrated and boiled (95°C, 30 min) in 0.01 M sodium citrate buffer. After blocking of Fc receptors with PBS containing 5% goat serum, sections were incubated with anti-ERK5 rabbit polyclonal antibody (Santa Cruz Biotechnology, Santa Cruz, CA) overnight at 4°C. Immunostaining was revealed using peroxidase-conjugated anti-rabbit or anti-mouse secondary antibody (Jackson ImmunoResearch, West Grove, Pennsylvania) and diaminobenzidine chromogen (Dako, Carpinteria, CA) as a substrate. Sections were counterstained with hematoxylin (Vector, Burlingame, CA). Mounting solution (Dako) and cover slips were added to the sections.

### BrdU staining

Proliferating C4-2, LNCaP and PNT2 cells were incubated for 6 h with BrdU. Cells grown on coverslips were fixed with 4% formaldehyde in PBS for 15 min at 4°C and then washed in PBS and permeabilized for 10 min in 0.25% Triton X-100 in PBS. After permeabilization, DNA was denaturated in 2N HCl for 10 min, and cells were incubated with blocking buffer (PBS-1% BSA). BrdU was then detected with the anti-BrdU monoclonal antibody (Dako, Carpinteria, CA) and visualized with a FITC anti-mouse secondary antibody (Jackson ImmunoResearch, West Grove, PA).

### In situ hybridization

LNA-modified probes (Exiqon) were 3′-end labeled with digoxigenin-ddUTP with terminal transferase using the Dig-3′-end labeling kit (Roche Diagnostics, France). 5 µm-thin sections were depleted of paraffin with xylene and rehydrated in a serial dilution of ethanol (2×100%, 75%, 50%, 25%). Slides were washed with PBS, digested with proteinase K (10 µg/ml) for 5 min at 37°C, rinsed in 0.2% glycine/PBS then in PBS, and postfixed with 10% formaldehyde in PBS (10 min). Slides were then rinsed with PBS (2 X). Slides were then prehybridized at 54°C for 2 hours in hybridization buffer (50% Formamide, 5xSSC, 0.1% Tween-20, adjusted to pH 6.0 with 9.2 mM Citric acid, 50 µg/ml heparin, 500 µg/ml yeast tRNA). Next, slides were hybridized in incubation chambers overnight at 54°C in an oven with constant motion, using 2 µl of probe in ∼200 µl of prewarmed hybridization buffer. Sections were rinsed twice in 2xSSC, followed by 3 washes of 30 min at 54°C in 50% formamide/2xSSC. Sections were then rinsed 5 times in PBS/0.1%Tween-20 (PBST), and blocked for 1 hr in blocking solution (2% sheep serum, 2 mg/ml BSA in PBST). Anti-digoxigenin antibody (Roche) was preadsorbed at 1∶1000 dilution in blocking solution for 2 hours and then applied on sections overnight at 4°C. Next, slides were washed 3 times in PBST and washed 2 times in AP buffer (100 mM Tris-HCl pH 9.5, 50 mM MgCl_2_, 100 mM NaCl, 0.1% Tween-20), followed by NBT/BCIP developing solution (50 ml Staining solution, 240 µl of 50 mg/ml NBT, 175 µl of 50 mg/ml BCIP, 1 mM Levamisole). The NBT/BCIP solution incubation usually was complete by one week. After color development, slides were rinsed in PBS, ddH2O and were dehydrated by passing through a series of alcohols (50%, 75%, 95%, 100%, 100%) and xylenes and coverslipped in Mounting Medium (Dako).

### Flow cytometry analysis of cell cycle and apoptosis

For cell cycle analysis, LNCaP or C4-2 cells were plated at a density of 2.5×10^5^ cells/dish in 6-wells dishes and transfected with either scrambled miR (control), miR 143 or antimiR 143. Cells were then rinsed in PBS, pelleted at 400 g for 5 min and maintained on ice for 20 min before resuspension in a 25 µg/ml propidium iodide (Sigma) solution. Cells were kept overnight at 4°C and the percentages of cells in G1, S and G2-M phases of the cell cycle were measured with a Coulter Epics XL™ flow cytometer (Becton Dickinson) using 488-nm laser excitation. For staining of late apoptotic and necrotic cells, cells were rinsed in PBS, pelleted at 400 g for 5 min and stained with propidium iodide (PI) at 1 ug/ml (Sigma) for 15 min at 4°C. The percentage of PI positive cells was immediately analyzed using 488–540-nm laser excitation. Figures show representative results of at least two independently performed experiments.For apoptosis experiment, cells were rinsed in PBS, pelleted at 400 g for 5 min and stained with annexinV-FITC (Roche Diagnostics, Meylan, france) for 15 min at 4°C. The percentage of annexin positive cells was immediately analyzed using 488-nm laser excitation. Figures show representative results of at least two independently performed experiments.

### Human active caspases-3 immunoassay

We used the active Caspase-3 Quantikine kit (R&D systems). Cells were transfected with miR-143, negative control miR. 48 h after transfection, cells are treated, as described by the manufacturer.

### Statistical analysis

Data are presented as means ± SEM. Group means were compared by factorial ANOVA. Differences were considered statistically significant at *P*<0.05.
